# Cyclosporine A in hospitalized COVID-19 pneumonia patients to prevent the development of interstitial lung disease: a pilot randomized clinical trial

**DOI:** 10.1038/s41598-024-54196-5

**Published:** 2024-02-15

**Authors:** Tatiana Cobo-Ibáñez, Gemma Mora Ortega, Carlos Sánchez-Piedra, Gonzalo Serralta-San Martín, Israel J. Thuissard-Vasallo, Vanesa Lores Gutiérrez, Llanos Soler Rangel, Cristina García Yubero, Ana Esteban-Vázquez, Elena López-Aspiroz, Cristina Andreu Vázquez, Inmaculada Toboso, Blanca María Martínez Alonso de Armiño, Rocío Alejandra Olivares Alviso, Rocío Calderón Nieto, Cecilia Yañez, Marlín Alejandra Zakhour González, Tatiana Sainz Sánchez, Silvia Arroyo de la Torre, Nazaret Del Amo Del Arco, Jorge Francisco Gómez-Cerezo, Teresa Ramírez Prieto, Alicia Martínez Hernández, Santiago Muñoz-Fernández

**Affiliations:** 1 Department of Rheumatology, Hospital Universitario Infanta Sofía, Universidad Europea de Madrid, 28702 Madrid, Spain; 2 Fundación para la Investigación e Innovación Biomédica del Hospital Universitario Infanta Sofía y Hospital Universitario del Henares (FIIB HUIS HHEN), 28702 Madrid, Spain; 3 Department of Pneumology, Hospital Universitario Infanta Sofía, Universidad Europea de Madrid, 28702 Madrid, Spain; 4 Instituto de Salud Carlos III, 28029 Madrid, Spain; 5 Department of Internal Medicine, Hospital Universitario Infanta Sofía, Universidad Europea de Madrid, 28702 Madrid, Spain; 6 Department of Medicine, Faculty of Biomedical and Health Sciences, Universidad Europea de Madrid, 28670 Madrid, Spain; 7 Deparment of Pharmacy, Hospital Universitario Infanta Sofía, 28702 Madrid, Spain; 8 Deparment of Immunology, Hospital Universitario Infanta Sofía, 28702 Madrid, Spain; 9 Department of Emergency, Hospital Universitario Infanta Sofía, 28702 Madrid, Spain; 10 Central Laboratory, Hospital Universitario Infanta Sofía, 28702 Madrid, Spain

**Keywords:** Diseases, Medical research

## Abstract

Post-COVID-19 interstitial lung disease (ILD) is a new entity that frequently causes pulmonary fibrosis and can become chronic. We performed a single-center parallel-group open-label pilot randomized clinical trial to investigate the efficacy and safety of cyclosporine A (CsA) in the development of ILD in the medium term among patients hospitalized with COVID-19 pneumonia. Patients were randomized 1:1 to receive CsA plus standard of care or standard of care alone. The primary composite outcome was the percentage of patients without ILD 3 months after diagnosis of pneumonia and not requiring invasive mechanical ventilation (IMV) (response without requiring IMV). The key secondary composite outcomes were the percentage of patients who achieve a response requiring IMV or irrespective of the need for IMV, and adverse events. A total of 33 patients received at least one dose of CsA plus standard of care (n = 17) or standard of care alone (n = 16). No differences were found between the groups in the percentage of patients who achieved a response without requiring IMV or a response requiring IMV. A higher percentage of patients achieved a response irrespective of the need for IMV in the CsA plus standard of care group although the RR was almost significant 2.833 (95% CI, 0.908–8.840; *p* = 0.057). No differences were found between the groups for adverse events. In hospitalized patients with COVID-19 pneumonia, we were unable to demonstrate that CsA achieved a significant effect in preventing the development of ILD. (EU Clinical Trials Register; EudraCT Number: 2020-002123-11; registration date: 08/05/2020).

## Introduction

Post-COVID-19 interstitial lung disease (ILD) is a new entity with a prevalence ranging from 21 to 44.9% depending on the length of follow-up and the radiological definition applied. It can lead to morbidity and mortality, disability, impaired quality of life, and associated costs^1–4^.

In SARS-CoV-2 infection, inflammatory immune cells, proinflammatory cytokines, and adhesion molecules are key factors in diffuse alveolar damage, which can progress to fibrosis^5,6^. Research efforts have been directed at testing the efficacy of agents capable of attenuating an immune response in the acute phase of the disease. In this regard, treatments used for autoimmune diseases, such as tocilizumab and bariticinib, have shown survival benefits in hospitalized patients with COVID-19 pneumonia^7,8^. In fact, a recent open-label, randomized, controlled trial suggested that baricitinib was non-inferior to tocilizumab with respect to mechanical ventilation or death by day 28 in hospitalized patients with severe COVID-19^9^. Therefore, investigation of other immunomodulatory agents such as cyclosporine A (CsA) seems reasonable. CsA and tacrolimus inactivate transcription of the *IL2* gene by inhibiting calcineurin phosphatase activity, thus suppressing T-lymphocyte proliferation^10^. In cell cultures, CsA and tacrolimus inhibit replication of SARS-CoV and expression of IL-2^11–13^. However, the doses administered are much higher than those recommended for use in humans. Therefore, these agents could exert an effect on COVID-19 by modulating the inflammatory response, and it remains unclear whether they also exert an antiviral effect.

This rationale has served as the basis for short-term studies to determine the effect of CsA and tacrolimus on COVID-19 pneumonia^14,15^. However, the efficacy of calcineurin inhibitors on medium-term outcomes, such as the development of post-COVID-19 ILD, is unknown. A treatment that could act on the inflammatory phase would prevent or reduce the likelihood of ILD, thus improving prognosis. Our hypothesis is that CsA could have a favorable effect on the immune response by acting as a disease modifier and thus improving the course of post-COVID-19 pneumonia and preventing medium-term outcomes such as ILD. In this randomized clinical trial, we evaluate the possible effect of CsA on the development of ILD in the medium term in patients hospitalized with COVID-19 pneumonia.

## Material and methods

### Trial design

We performed a single-center parallel-group open-label pilot randomized clinical trial (the CYCLO trial). The study was approved by the Ethics Committee of Hospital Universitario la Paz (code: 5588; May 6, 2020) and the Spanish Agency of Medicines and Medical Devices and registered in the EU Clinical Trials Register (https://www.clinicaltrialsregister.eu/ctr-search/trial/2020-002123-11/ES; EudraCT Number: 2020-002123-11; registration date: 08/05/2020). All procedures were in accordance with relevant guidelines and regulations. It is confirmed that informed consent was obtained from all subjects and/or their legal guardian(s). The study was performed at Hospital Universitario Infanta Sofía (Madrid, Spain). A clinical research associate was responsible for external monitoring of patient data and safety.

### Participants

To be included in the study patients had to be adults (≥ 18 to < 80 years) and hospitalized with symptoms of SARS-CoV-2 infection (fever and/or cough and/or dyspnea and/or myalgia and/or asthenia and/or diarrhea) for ≤ 7 days. They also had to have pulmonary infiltrates on their chest X-ray, baseline oxygen saturation < 95%, status ≥ 3 on the WHO ordinal scale (hospitalized with mild disease [no oxygen therapy, oxygen mask, or nasal prongs] or with serious disease [non-invasive mechanical ventilation, high-flow nasal cannula, invasive mechanical ventilation with or without additional support]) (see “Supplementary Material”), and a positive PCR result for SARS-CoV-2 (nasal and pharyngeal swab).

The exclusion criteria were as follows: contraindication or allergy to CsA, inability to provide informed consent, previous ILD, participation in other studies on COVID-19, treatment with prednisone or equivalent > 10 mg/d, treatment with IL-1 or IL-6 inhibitors during the previous 30 days, treatment with rituximab during the previous 6 months, treatment with other biologics or immunoglobulins during the previous 2 months, kidney failure with an estimated glomerular filtration rate < 30 mL/min, poorly controlled arterial blood pressure, active infection, AST/ALT values > 5 times the upper limit of normal, neutrophils < 500/mm^3^, platelets < 50,000/mm^3^, active neoplasm, pregnancy, breastfeeding, and major comorbidity (Cumulative Illness Rating Scale > 29, see “Supplementary Material”).

Patients were selected in the emergency department while they waiting to be admitted to hospital or after hospitalization. Informed consent was obtained before randomization. The visits were in the ward and, after discharge, in the hospital clinic.

### Randomization

Eligible patients who signed the informed consent document were randomly assigned to the CsA-standard of care (SOC) group (intervention group) or the SOC group (control group). The computer-generated randomization list was obtained by an independent investigator using the Research Randomizer program^16^. The allocation was 1:1 based on a random block size of 4. The allocation sequence was concealed from the investigators enrolling the patients. The investigators were only informed of the intervention assigned to each patient.

### Blinding

Open-label study. The patients, physicians, and study staff were aware of the treatment assigned. The pharmaceutical presentation and route of administration for CsA, as well as the absence of a plausible alternative as placebo, precluded blinding.

### Intervention

In the study group, CsA was administered orally (capsules or oral solution if the patient was unable to swallow the capsules), with the total dose divided into 2 doses per day. The initial dose was as follows: < 60 kg, 100 mg/d; 60–80 kg, 150 mg/d; and > 80 kg, 200 mg/d. At 48 h, the dose was adjusted to the following: < 60 kg, 150 mg/d; 60–80 kg, 200 mg/d; and > 80 kg, 300 mg/d. Trough blood or plasma levels were monitored every 48 h until the therapeutic margin of 100–250 ng/mL was reached. Depending on the levels, the dose could be increased or decreased by 50 mg/d up to a maximum of 5 mg/kg/d. Furthermore, the dose could be adjusted within the therapeutic margin if a ≥ 30% increase was detected in the baseline creatinine value or in the case of poor control of arterial blood pressure. The duration of treatment with CsA was 1 month.

### Concomitant treatment

Concomitant treatment includes treatments that were administered to both groups. Indications, dose, and duration are specified in the “Supplementary Material”.

SOC followed national recommendations (AEMPS: www.aemps.gob.es/la-aemps/ultima-informacion-de-la-aemps-acerca-del-covid%e2%80%9119/) and international recommendations (WHO: www.who.int/health-topics/coronavirus#tab=tab_1), which are regularly updated. Furthermore, rescue treatment was with methylprednisolone (250 mg iv every 24 h for 3 days, followed by 40 mg every 12 h for 2 days, 40 mg every 24 h for 2 days and suspension) or biological therapy (tocilizumab iv, single dose of 600 mg for ≥ 75 kg and 400 mg for < 75 kg; sarilumab iv, single dose of 400 mg; or anakinra sc, 100 mg/6 h for 3 days).

### Data collection

An online case report form was designed to collect sociodemographic, clinical, laboratory, and radiologic data at baseline (day 1), day 4, day 8, at discharge day 30, and day 90, or when an adverse event was recorded, or until the patient died. The online case report form was tested in a pilot study with data from 2 hypothetical cases before initiation of the study. Inconsistencies in data entry were recorded.

### Outcomes

The primary composite outcome was a response without requiring invasive mechanical ventilation (IMV) at 3 months after the diagnosis of COVID-19 pneumonia. This was defined as the percentage of living patients who had not developed ILD and did not require IMV during hospitalization. ILD was defined by radiological findings at 3 months after the diagnosis of COVID-19 pneumonia. Radiologic findings were the presence of traction bronchiectasis, subpleural parenchymatous bands, and/or a reticular pattern on the computed tomography scan. Computed tomography scans (CTS) were evaluated by a single radiologist with expertise in ILD.

The secondary outcomes were as follows: (a) response requiring IMV (percentage of living patients who did not develop ILD by radiological findings and required IMV during admission) at 3 months after the diagnosis of COVID-19 pneumonia; (b) response irrespective of the need for IMV (percentage of living patients who did not develop ILD by radiological findings and required and did not require IMV during admission) at 3 months after the diagnosis of COVID-19 pneumonia; (c) percentage of patients who did not achieve a response at 3 months after the diagnosis of COVID-19 pneumonia, either because they had developed ILD by radiological findings or died; (d) percentage of patients who required IMV, methylprednisolone, or biologics; (e) time between diagnosis and IMV, methylprednisolone, or biologics; (f) percentage of adverse events at 3 months after the diagnosis of COVID-19 pneumonia. Adverse event was defined as any incident detrimental to health that occurs in the clinical trial subjects, even if it does not necessarily have a causal relationship with it.

Other secondary outcomes included the following: (a) patient progress according to the WHO ordinal scale and ratio of oxygen saturation by pulse oximetry to the fraction of inspired oxygen (SpFi); (b) negative PCR result for SARS-CoV-2 at discharge; (c) development of antibodies, pulmonary function test (PFT) results, and percentage of ILD at 3 months after diagnosis of COVID-19 pneumonia.

### Sample size

This study was designed as a pilot randomized clinical trial. A sample size was not set a priori.

### Statistical analyses

The full analysis set included all randomized patients who received at least one dose of treatment.

In the descriptive analysis, qualitative variables were expressed as absolute frequencies (n) and relative frequencies (%), together with the 95% confidence interval (CI). Normally distributed quantitative variables were expressed as mean and standard deviation (Shapiro–Wilk test); non-normally distributed variables were expressed as median and interquartile range (Q25–Q75).

The variables were compared between the study group and the control group using the independent-samples *t* test in the case of those following a normal distribution; otherwise, the Mann–Whitney test was used. Qualitative variables (nonordinal) were studied using the chi-squared test or Fisher exact test.

We estimated the percentage of responders and non-responders and the need for IMV, methylprednisolone, or biologics (with 95% CI). We also determined the relative risk (RR) with its 95% CI for the above-mentioned variables.

Time between diagnosis and IMV, methylprednisolone, biologics or adverse events was studied using a Kaplan–Meier survival analysis and the Mantel–Haenszel statistic (log-rank test). The result was expressed as the hazard ratio (HR) with its 95% CI. Changes in the ordinal scale of the WHO and SpFi were measured using the Friedman test (parametric) after verifying the normal distribution of the values recorded at the various time-points.

All statistical tests were 2-sided, and p values below 0.05 were considered statistically significant. The statistical analyses were performed using IBM SPSS (Version 25.0, IBM Corp, Armonk, NY, USA).

## Results

### Patients

**Figure 1:**
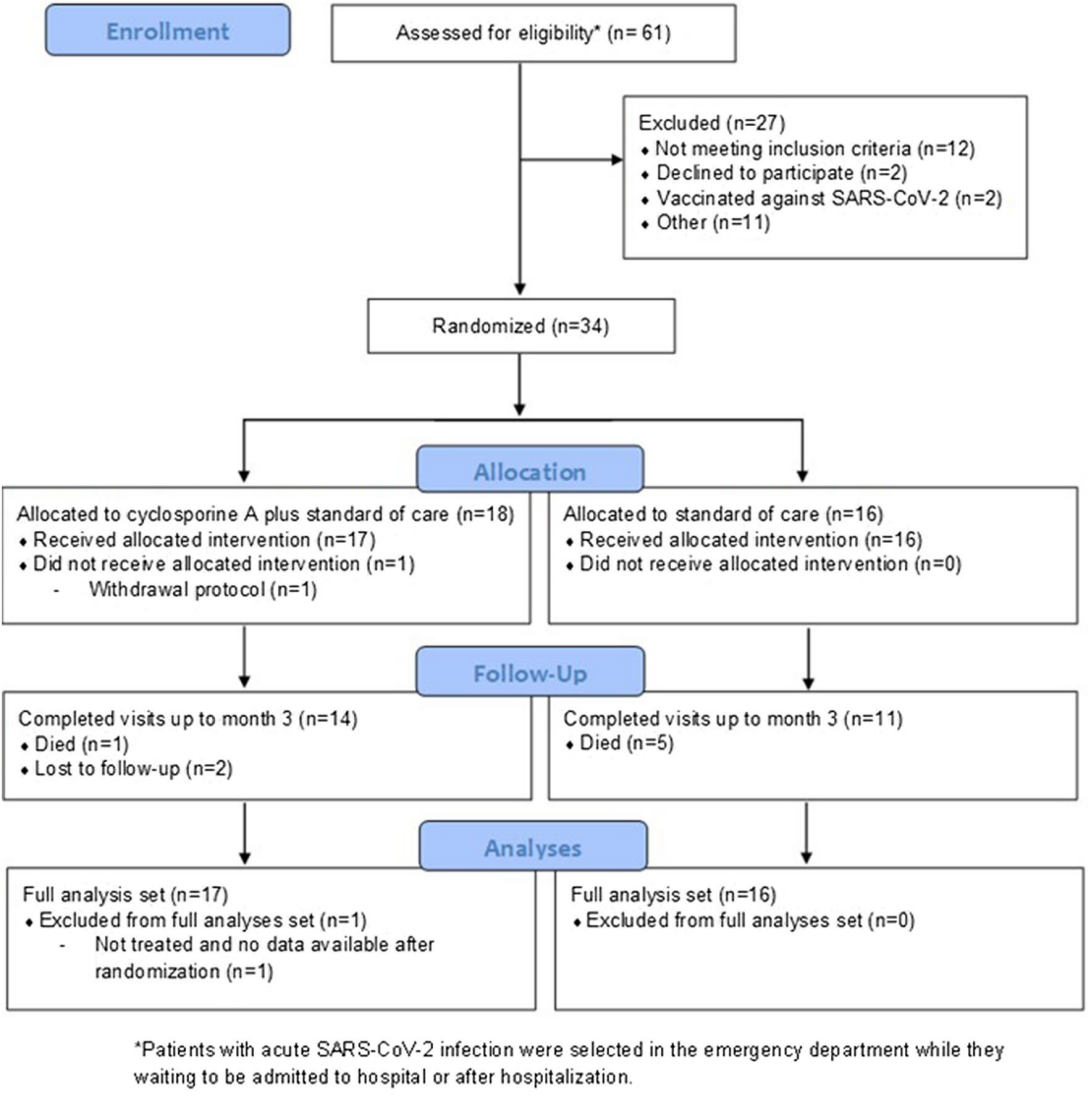
Enrollment, randomization, and follow-up.

A total of 61 patients were selected to be included in the trial. Thirty-four were eventually assigned to CsA-SOC (n = 18) or SOC (n = 16). One patient from the CsA group was excluded from the full analysis set because of admission to the intensive care unit after randomization. Therefore, the patient did not receive the treatment assigned (Consort diagram, Fig. 1).

The recruitment period started on May 12, 2020 and ended on May 15, 2021. The follow-up period ended on September 21, 2021.

Mean age was 56.7 ± 11.8 years, 33.3% were women, and 69.7% were Caucasian. The baseline characteristics were similar between the study groups, except for temperature and white blood cell count (neutrophils), which were higher in the CsA-SOC group (Table 1).

**Table 1:** Patient Baseline Characteristics*.

Characteristic	Total (N = 33)	Cyclosporine A plus standard of care (N = 17)	Standard of care (N = 16)
Sociodemographic			
Age years	56.7 ± 11.8	53.6 ± 12.1	60.1 ± 11.0
Female sex	11 (33.3)	5 (29.4)	6 (37.5)
Race***			
Caucasian	23 (69.7)	11(64.7)	12(75.0)
Hispanic	8 (24.2)	6 (35.3)	2 (12.5)
African (Black and North-African)	2 (6.0)	0 (0.0)	2 (12.5)
Education			
None or basic	19 (57.5)	10 (52.6)	9 (47.4)
Middle or high	14 (42.4)	7 (50.0)	7 (50.0)
Comorbidities**			
Body mass index	28.3 [25.8–36.7]	28.3 [27.7–35.0]	28.4 [25.5–36.7]
Arterial hypertension	9 (27.2)	7 (41.2)	2 (12.5)
Diabetes mellitus	6 (18.1)	3 (17.6)	3 (18.8)
Cerebrovascular disease	1(3.0)	0 (0.0)	1(6.3)
Former or active smoker	11 (33.3)	5 (29.4)	6 (37.5)
ACE inhibitors	4 (12.1)	4 (23.5)	0 (0.0)
ARA II	4 (12.1)	3 (17.6)	1 (6.2)
Clinical status			
Temperature (°C)	36.8 [36.3–37.7]	37.4 [36.5–38.0]	36.5 [36.3–36.9]
Respiratory rate (bpm)	20 [18.0–24.0]	22 [18.0–24.0]	20 (17.0–23.5]
Blood oxygen saturation (0–100)	90.0 [88.0–92.5]	91.0 [90.0–92.0]	89.5 [ 88.0–92.5]
SpFi (0- > 600)	346.0 [212.5- 423.5]	344.0 [267.0–396.0]	411.5 [182.0–433.0]
SOFA score (0–24)	1 [0.0–2.5]	1 [0.0–2.0]	2 [0.0–3.5]
qSOFA score (0–3)	0 [0–1]	0 [0–1]	0 [0–1]
CURB-65 score (0–5)	0 [0–1]	0 [0–1]	1 [0–1]
WHO ordinal scale (0–8)	4 [4.00–4.05]	4 [4.00–4.00]	4 [4.00–5.00]
Laboratory parameters			
IL-6 (pg/mL)	20.10 [5.65–39.95]	24.1 [9.20–35.90]	11.65 [5.50–36.35]
White blood cell count (cells/mm^3^)	6610 [5235–8970]	8590 [6330- 10980]	5875 [4895–6850]
Neutrophils (cells/mm^3^)	5000 [3750–6750]	6300 [4600- 7500]	4300 [3200–5000]
Lymphocytes (cells/mm^3^)	900 [600–1150]	900 [700–1200]	900 [550–1100]
CRP (mg/L)	87.5 [36.4–126.2]	77.7 [31.2–117.6]	108.4 [47.2–128.2]
D-dimer (ng/ml)	490 [325–760]	430[320–540]	590 [455–945]
Ferritin (ng/mL)	772 [362.0–1250.0]	829 [556.5–1278.0]	71 [183.0–1076.0]
Troponin 1 (ng/mL)	0.02 [0.02–0.02]	0.02 [0.02–0.02]	0.02 [0.02–0.02]
LDH (U/L)	313.0 [272.5–394.0]	274.0 [264.0–353.0]	356.5 [300.0–402.0]
Procalcitonin (ng/mL)	0.060 [0.020–0.110]	0.065 [0.035–0.095]	0.060 [0.020–0.120]
Chest X-ray			
Unilateral infiltrates	2 (6.0)	1 (5.8)	1 (6.2)
Bilateral infiltrates	29 (87.8)	15 (88.2)	14 (87.5)
Diffuse infiltrates	2 (6.0)	1 (5.8)	1 (6.2)
Standard of care			
Third-generation cephalosporin	33 (100)	17 (100)	16 (100)
Low-molecular-weight heparin	33 (100)	17 (100)	16 (100)
Remdesivir	12 (36.4)	7 (41.2)	5 (31.3)
Dexamethasone	31 (93.9)	17 (100)	14 (87.5)

Data are shown as n (%), mean ± standard deviation, and median [interquartile range].

*No differences between groups except for temperature (*p* = 0.017), white blood cell count (*p* = 0.009), and neutrophils (*p* = 0.011).

**No patients diagnosed with cardiovascular disease, chronic obstructive pulmonary disease, chronic kidney failure, chronic liver disease, or cancer. No patients receiving immunosuppressive treatment.

***Not calculable.

ACE inhibitors: angiotensin-converting enzyme inhibitors; ARAII: angiotensin II receptor antagonist; ºC: degrees centigrade; bpm: breaths per minute; SpFi: ratio of oxygen saturation by pulse oximetry to the fraction of inspired oxygen; SOFA: Sequential Organ Failure Assessment; qSOFA: quick SOFA; CURB-65: Confusion-Uremia-Respiratory rate- Blood pressure-age > 65; WHO: Word Health Organization; IL-6: interleukin 6; CRP: C-reactive protein; LDH: lactate dehydrogenase.

### Primary outcome

No differences were found between the groups with respect to achieving a response without requiring IMV, although a higher percentage of patients achieved a response without requiring IMV in the CsA-SOC group than in the SOC group (76.5% [95% CI, 56.3–96.7] vs. 50% [95% CI, 25.5–74.5]; *p* = 0.114). While the probability of response without requiring IMV in the CsA-SOC group was greater, the difference was not significant (RR, 3.250 [95% CI, 0.733–14.402]; *p* = 0.121) (Table 2).

**Table 2:** Primary and secondary outcomes at 3 months after the diagnosis of COVID-19 pneumonia.

Full analysis set (n = 33)	Estimate (95% CI) or [interquartile range]		*p*-value	Treatment effect	*p*-value
	Cyclosporine A plus standard of care (n = 17)	Standard of care (n = 16)		Estimate (95% CI)	
Primary outcome					
Response without requiring IMV	76.5 (56.3–96.7)	50 (25.5–74.5)	0.114	RR: 3.250 (0.733–14.402)	0.121
Secondary outcomes					
Response requiring IMV	5.9 (0.0–17.1)	0 (0–0)	1.000	RR: NC	NC
Response irrespective of need for IMV	82.4 (64.3–100.0)	50 (25.5–74.5)	0.049	RR: 2.833 (0.908–8.840)	0.057
No response due to ILD*	12.5 (0–28.2)	27.3 (5.5–49.1)	0.370	RR: 0.381 (0.052–2.784)	0.342
No response due to death	5.9 (0–17.1)	31.3 (8.4–53.8)	0.085	RR: 0.138 (0.014–1.344)	0.342
Need for IMV	11.8 (0–27.1)	37.5 (13.8–61.2)	0.118	RR: 0.138 (0.014–1.344)	0.088
Need for MEP	52.9 (29.2–76.6)	56.3 (32.0–80.6)	0.849	RR: 0.222 (0.037–1.330)	0.099
Need for biological therapy	17.6 (0–35.7)	12.5 (0–28.7)	1.000	RR: 0.875 (0.222–3.451)	0.849
Days to IMV	5.5 [4.0–7.0]	7.5 [3.0–11.0]	0.866	HR: 0.300 (0.073–1.223)	0.093
Days to MEP	3 [3, 4]	2 [0–3]	**0.029**	HR: 1.102 (0.359–3.384)	0.864
Days to biological therapy	10 [4–10]	5 [2–8]	0.236	HR: 1.352 (0.230–7.921)	0.738
Days to MEP or biological therapy	3 [3, 4]	2 [0–3]	0.029	HR: 1.102 (0.359–3.384)	0.864
Safety outcomes					
Adverse events	11.8 (0.0–27.1)	6.3 (0.0–18.2)	1.000	RR: 2.000 (0.163–24.484)	0.565
Days to adverse events	11.5 [NC-NC]	18 [NC-NC]	NC	HR: 1.869 (0.019–17.980)	0.588

Data are shown as rate % (95% confidence interval), median [interquartile range], risk ratio (95% confidence interval), and hazard ratio (95% confidence interval).

*CI* confidence interval; *RR* risk ratio; *NC* not calculable; *ILD* interstitial lung disease; *IMV* invasive mechanical ventilation; *MEP* methylprednisolone; *HR* hazard ratio.

*Denominator of 16 and 11 in cyclosporine A-standard of care group and in standard of care group, respectively. No data at 3 months on interstitial lung disease because the patients had died (6 patients).

Response without requiring IMV: The percentage of living patients whose interstitial lung disease did not persist 3 months after diagnosis of COVID-19 pneumonia and who did not require IMV during hospitalization.

Response requiring IMV: The percentage of living patients whose interstitial lung disease did not persist 3 months after diagnosis of COVID-19 pneumonia and who required IMV during hospitalization.

Response irrespective of the need for IMV: The percentage of living patients whose interstitial lung disease did not persist 3 months after diagnosis of COVID-19 pneumonia and who required and did not require IMV during hospitalization.

### Secondary outcomes

No differences were found between the groups with respect to achieving a response requiring IMV, although a higher percentage of patients achieved a response requiring IMV in the CsA-SOC group than in the SOC group (5.9% [95% CI, 0.0–17.1%] vs. 0% [95% CI, 0–0%]; *p* = 1.000) (Table 2).

A higher percentage of patients achieved a response irrespective of the need for IMV in the CsA-SOC group than in the SOC group (82.4% [95% CI, 64.3–100.0%] vs. 50% [95% CI, 25.5–74.5%]; *p* = 0.049) although the effect of CsA was not significant (RR, 2.833 [95% CI, 0.908–8.840]; *p* = 0.057) (Table 2). The number needed to treat was 3.

No differences were found between the groups on the development of ILD, death or need for IMV or methylprednisolone, although the percentage of patients was lower in the CsA-SOC group than in the SOC group (Table 2). No differences were found between the groups in relation to biologics received, although the percentage of patients was higher in the CsA-SOC group than in the SOC group (Table 2). Tocilizumab was the only drug used.

There were no differences between the groups in the development of ILD, death or the need for IMV or methylprednisolone.

**Figure 2:**
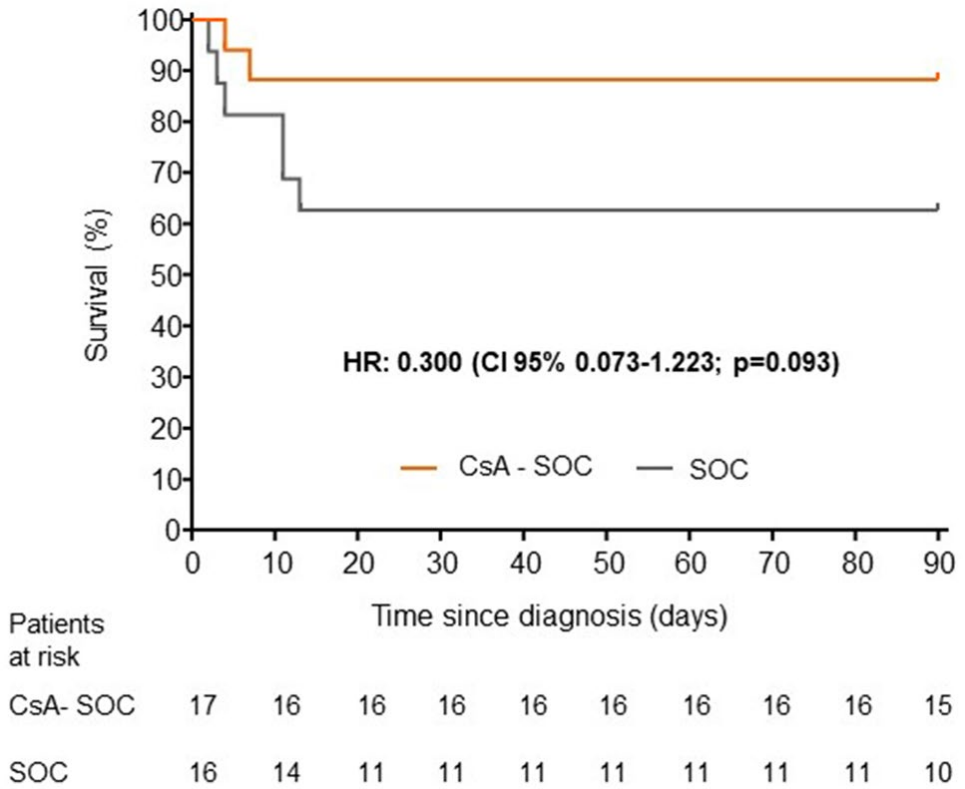
Time from diagnosis to invasive mechanical ventilation. HR: hazard ratio; CI: confidence interval; CsA: cyclosporine A; SOC: standard of care.

The median number of days until methylprednisolone was required was greater in the CsA-SOC group than in the SOC group (3 [3–4] vs. 2 [0–3]; *p* = 0.029), with no differences between the groups in days until IMV or biologics (Table 2). There were no statistically significant differences between groups in the time from diagnosis to non-invasive mechanical ventilation (Fig. 2, Table 2). There were also no statistically significant differences between groups in the time from diagnosis to the need for methylprednisolone or biological therapy (Figs. 1 and 2 in the Supplementary Appendix, Table 2).

### Other outcomes

Among the 25 patients who attended their visit at 3 months, we detected a clinical improvement, with differences in the ordinal scale of the WHO and in SpFi between days 1 and 30 and days 1 and 90 (*p* < 0.001). Progress was also better for SpFi in the CsA-SOC group than in the SOC group, with no differences between the groups in the WHO ordinal scale (Table 3). SARS-CoV-2 PCR results returned to negative values in 76% of patients at discharge, with no differences between the groups (Table 3). All patients developed IgG against SARS-CoV-2.

**Table 3:** Differences between groups in the WHO ordinal scale, SpFi, and SARS-Cov-2 PCR result at 3 months after the diagnosis of COVID-19 pneumonia.

	Total (n = 25)	Cyclosporine A plus standard of care (n = 14)	Standard of care (n = 11)	*p*-value
WHO ordinal scale				
Day 1	4 [4–4]	4 [4–4]	4 [4, 5]	0.289
Day 30	0 [0–0]	0 [0–0]	0 [0–6]	
Day 90	0 [0–0]	0 [0–0]	0 [0–0]	
SpFi				
Day 1	346.0 [313.5–423.5]	343.5 [259.5–368.3]	419.0 [346.0–438.0]	0.037
Day 30	457.0 [452.0–461.0]	457.0 [452.0–466.0]	457.0 [450.0–461.0]	
Day 90	461.0 [454.5–466.0]	461.0 [452.0–466.0]	461.0 [457.0–466.0]	
Negative SARS-CoV-2 PCR test at discharge	19 (76.0)	11 (78.6)	8 (72.7)	0.734

Data are shown as median [interquartile range] and n (%).

*WHO* Word Health Organization; *SpFi* ratio of oxygen saturation by pulse oximetry to the fraction of inspired oxygen; *SARS-CoV-2* severe acute respiratory syndrome coronavirus 2; *PCR* polymerase chain reaction.

The 25 patients who attended their 3-month visit and the 2 patients from the CsA-SOC group who were lost to follow-up had PFT results within the reference range, with no differences between the groups (Table 4). No differences in the development of ILD were found between the groups.

**Table 4:** Differences between groups in pulmonary function tests at 3 months after the diagnosis of COVID-19 pneumonia.

	Total (n = 27) *	Cyclosporine A plus standard of care (n = 16)	Standard of care (n = 11)	*p*-value
FVC (mL)	3250 [2650–3720]	3450 [2805–3885]	3130 [1990–3480]	0.372
FVC (% of predicted value)	92 [75–102]	87 [77–95]	101 [73–105]	0.336
FEV_1_ (mL)	2770 [ 2090–3110]	2815 [2145–3405]	2730 [1470–3050]	0.509
FEV_1_ (% of predicted value)	93.0 [72.0- 104.0]	90.0 [75.5–96.0]	104.0 [25.0–75.0]	0.270
FEV_1_/FVC	82 [80–87]	82 [80–87]	85 [80–87]	0.555
DLCO (% of predicted value)	95 [85–110]	91 [84–109]	105 [85–114]	0.847

Data are shown as median [interquartile range].

*FVC* forced vital capacity; *FEV*_*1*_ forced expiratory volume in one second; *DLCO* diffusing capacity of the lung for carbon monoxide.

*Visits were completed by 25 patients up to month 3 plus 2 patients in the cyclosporine A plus standard of care group that were lost to follow up with pulmonary function data at 3 months after the diagnosis of COVID-19 pneumonia.

No differences in the development of ILD were found between the groups, although the percentage of patients was lower in the CsA-SOC group than in the SOC group (2/16, [12.5%; 95% CI, 0.0–31.2%] vs. 3/11 [27.3%, 95% CI, 2.1–52.5%]; *p* = 0.307). The PFT results revealed a restrictive pattern in 1/2 and 2/3 patients, respectively.

### Safety outcomes

A higher percentage of patients experienced adverse events in the CsA-SOC group than in the SOC group (11.8% [95% CI, 0–27.1%] vs. 6.3% [95% CI, 0–18.2%]), with no significant differences (*p* = 1.000) (Table 2). One patient in the CsA-SOC group had genital (type 2) herpes simplex infection and another had hypoglycemia. One patient in the SOC group had nosocomial bacterial pneumonia. There were no significant differences between groups in the time from diagnosis to adverse event (Fig. 3 in the Supplementary Material, Table 2).

## Discussion

In this parallel-group pilot randomized clinical trial, we analyzed the effect of CsA on the development of ILD after 3 months in patients hospitalized with COVID-19 pneumonia. No significant differences were found between the groups with respect to the percentage of patients without ILD who did not need IMV, although the number of patients was higher in the CsA-SOC group. In this group, CsA tended to increase the percentage of patients without ILD when both patients requiring IMV and patients not requiring IMV during admission were included in the analysis.

Pfefferle et al.^11^ used cell cultures to demonstrate that nonstructural protein 1 (Nsp1) from several CoVs included in SARS-CoV interacts with various immunophilins. They also showed that Nsp1 induces expression of IL-2, which is involved in the deregulation of SARS-CoV cytokines. In T lymphocytes, immunophilins such as cyclophilin and FK506-binding proteins (FKBP) favor the calcineurin phosphatase activity that promotes migration of NF-AT from the cytoplasm to the nucleus, where it activates transcription of the *IL2* gene, leading to production of this interleukin. IL-2 is necessary for progression of the different phases of the cell cycle in T lymphocytes and, therefore, for their proliferation and differentiation^10^. CsA and tacrolimus inhibit calcineurin phosphatase activity by means of the cyclosporine-cyclophilin complex and the tacrolimus-FKBP-calmodulin-calcineurin complex, respectively. Consequently, they suppress proliferation of T lymphocytes and, based on this effect, have been used to prevent rejection of a transplanted organ and for the treatment of various immune-mediated diseases. CsA inhibits replication of SARS-CoV in cell cultures and the expression of IL-2 favored by Nsp1^11,12^. Thus, we might expect, at least, an immunomodulatory effect of CsA in SARS-CoV-2 infection.

Post-COVID-19 ILD has been reported, both in patients who required hospital admission and in outpatients^3,17^. We were unable to find studies analyzing the efficacy of calcineurin inhibitors in reducing the development of post-COVID-19 ILD. Of note, however, a subgroup of idiopathic inflammatory myopathies, known as clinically amyopathic dermatomyositis (CADM), are associated with antibodies against melanoma differentiation–associated protein 5 (MDA5), which is an RLR helicase and putative cytoplasmic receptor for SARS-CoV2. Patients with CADM and anti-MDA5 antibodies can develop a syndrome comprising rapidly progressive ILD and refractory respiratory insufficiency similar to that generated by SARS-CoV-2. The condition of affected patients can improve when a calcineurin inhibitor is administered early during the disease course^18,19^. Our study revealed that a higher percentage of patients had not developed ILD at 3 months in the CsA-SOC group, irrespective of the need for IMV during admission, although the RR was not significant (2.833 [0.908–8.840]; *p* = 0.057). The percentage of patients requiring IMV was lower in the CsA-SOC group, although the differences between the groups were not sufficient to meet the primary objective (no ILD and no IMV).

The few published studies on calcineurin inhibitors for treatment of COVID-19 pneumonia focus on short-term outcomes. A single-center retrospective study (n = 607) revealed that of all the treatments used for COVID-19 pneumonia (corticosteroids, lopinavir/ritonavir, hydroxychloroquine, tocilizumab, and CsA), only CsA was associated with a significant decrease in in-hospital mortality^20^. A nonrandomized interventional study (n = 209) comparing CsA combined with low doses of corticosteroids and low doses of corticosteroids for 7–10 days showed that CsA reduced in-hospital mortality and led to an improvement in symptoms at 28 days after diagnosis of moderate-severe pneumonia^14^. In a phase I clinical trial without comparator, CsA was administered to 10 patients for a median of 4 days. During the 30-day follow-up, symptoms improved, and a decrease was recorded in levels of CXCL10, which has been associated with greater viral load, respiratory insufficiency, pulmonary lesions, and mortality in SARS-CoV-2 infection. A significant reduction was also observed in type I IFN gene expression signatures associated with exacerbated hyperinflammation in the peripheral blood cells of patients with COVID-19^21^. Another observational study with 20 patients revealed no benefits in terms of mortality, intensive care unit stay, and hospitalization, although data from only 2 days were analyzed^22^. A phase 2, single-center, randomized clinical trial involving 55 patients followed for 56 days and treated with tacrolimus + methylprednisolone pulses + SOC vs. SOC revealed a decrease in mortality, although the difference was not significant^15^. The results of our study were favorable for similar outcomes despite differences in design, SOC, and follow-up at 3 months. The mortality rate was lower in the CsA-SOC group, albeit without significant differences. We recorded differences in the improvement in SpFi over 3 months in CsA-SOC vs. SOC and found that the CsA-SOC group took longer (days) before requiring methylprednisolone. Given that the optimal dose for COVID-19 pneumonia is unknown, we decided not to increase the dose of CsA above 5 mg/kg/d for 30 days in order to obtain an effect in the medium term. This is the dose we administer in clinical practice. It is recommended both for treatment of immune-mediated processes for which CsA is indicated in the summary of product characteristics (nephrotic syndrome, psoriasis, rheumatoid arthritis, atopic dermatitis, uveitis) and for ILD associated with idiopathic inflammatory myopathy^18,23^. Higher doses can increase the risk of kidney failure. Elsewhere, the dose varied from 1 to 10 mg/kg/d over 2 to 21 days^14,15,20–22^. While we do not know whether the usual doses of CsA can have an antiviral effect in humans, there may have been a synergetic effect in patients who received remdesivir in our study. We recorded 2 adverse events in the intervention group (genital [type 2] herpes simplex and hypoglycemia) and 1 in the placebo group (bacterial superinfection), with no significant differences between the groups. None of the adverse effects reported were severe, as with the other studies.

Our study was subject to a series of limitations. The randomized clinical trial was not blinded, although both groups received similar concomitant treatments and had similar baseline characteristics, except for higher temperature and higher white blood cell count in the intervention group. In addition, the primary outcome was an objective outcome (without requiring IMV) and therefore not subject to interpretation by the investigator, patient, or statistician. The lack of sample size calculation in advance was a limitation, so the results should be interpreted with caution. Furthermore, the sample size was small because the study was performed at a single-center, and it was difficult to recruit patients with symptoms for ≤ 7 days who fulfilled the inclusion criteria at the beginning of the pandemic. The small sample size may be the reason the primary outcome was not reached; in addition, the definition of the outcome was too demanding, since it included absence of the need for IMV. The study was designed at the start of the pandemic, and we believed that CsA could obviate the need for IMV. However, experience shows that COVID-19 pneumonia can worsen in a few hours, often necessitating IMV early. When the need for IMV was eliminated, the percentage of response irrespective of the need for IMV at 3 months was higher in the CsA-SOC group than in the SOC group, although the RR was not significant (2.833 [0.908–8.840]; *p* = 0.057). A larger sample would be necessary to confirm with sufficient statistical power the association between CsA and the percentage of response irrespective of the need for IMV. Tarraso et al. observed post-COVID-19 pulmonary fibrotic changes in patients who do not require IMV and considered these a consequence of the viral infection itself^24^. The authors did not find IMV to be associated with pulmonary fibrosis 1 year after hospitalization for COVID-19. Therefore, we believe that it is beneficial not to develop ILD at 3 months, irrespective of whether IMV was necessary during admission. Another limitation was that CTS were not evaluated by two independent radiologists. It was recently suggested that higher plasma levels of IL-1α and TGF-β and lower levels of IFN-β could point to a greater risk of changes similar to those observed in pulmonary fibrosis in patients after COVID-19^17^. We did not assess this type of biomarkers in this pilot clinical trial.

The design of our study improves on that of previous studies with CsA. Neither development of ILD nor the remaining outcomes have previously been analyzed in the medium term. Since post-COVID-19 ILD has been reported to resolve after the acute process^25^, detecting it at 3 months makes it less likely to be overestimated. Furthermore, we define ILD by changes similar to fibrosis, with a lower probability of reversibility. However, this was an exploratory trial with a small sample and inconclusive results that must be confirmed. Several improvements could be made to ensure that our approach is more easily applicable in future trials. In addition to the study being blinded and multicenter, the outcome of ILD should not be limited by the need for IMV. Machine learning radiographic models could be used for accurate radiographic assessment of post-COVID-19 ILD^26^. Similarly, biomarkers of post-COVID-19 pulmonary fibrosis should be measured at baseline and during follow-up to determine how they change with treatment and their association with fibrosis. Several studies analyzing the prevalence and predictors of the development or progression of post-COVID-19 ILD have been conducted in hospitalized patients at 12 months post-infection^27,28^. In future trials, outcomes should be assessed in the long term (12 months) to ensure detection of post-COVID-19 ILD. Standard of care should include antiviral agents. While administration of CsA requires assessment of levels to ensure safety, the drug is inexpensive and widely used in clinical practice for treatment of chronic immune-mediated diseases in the medium-to-long term. Moreover, it is currently easier to detect SARS-CoV-2 infection and, therefore, to include patients with a short history of symptoms (days) in future clinical trials.

While the incidence of SARS-CoV-2 infection has decreased owing to measures such as vaccination, hospital admissions for COVID-19 pneumonia continue. Our preliminary results seem to indicate—but do not prove—that CsA has a beneficial effect post-COVID-19 ILD. It is necessary to develop a research line assessing CsA, taking into account the improvements proposed with respect to this pilot clinical trial. We should thus be able to demonstrate the efficacy and safety of calcineurin inhibitors in medium-to-long term outcomes such as ILD.

In conclusion, in patients hospitalized with COVID-19 pneumonia, we were unable to demonstrate that CsA has a significant effect on the development of ILD. However, CsA tended to increase the percentage of patients without ILD at 3 months after diagnosis when patients requiring and not requiring IMV during admission were considered. It is important to note that the present trial was exploratory and that we identified areas for improvement.

### Supplementary Information

Supplementary Information.

## Data Availability

The original contributions presented in the study are included in the article, further inquiries can be directed to the corresponding author.
