# Supplementary material for: Cyclosporine A in hospitalized COVID-19 pneumonia patients to prevent the development of interstitial lung disease: a pilot randomized clinical trial
Source: Sci Rep. 2024 Feb 15;14:3789. doi: 10.1038/s41598-024-54196-5 (PMC10869838; doi:10.1038/s41598-024-54196-5)
Supplement: Supplementary file 1 — Supplementary Information. [file 41598_2024_54196_MOESM1_ESM.docx]

Supplementary Material

**Table of contents**

## Patient status according to the WHO ordinal scale.

## Cumulative illness rating score.

## Concomitant treatments.

- Figures.

## CONSORT 2010 checklist of information to include when reporting a pilot or feasibility trial.

# Patient status according to the WHO ordinal scale

| **Patient status** | **Descriptor** | **Value** |
| --- | --- | --- |
| Not infected | No clinical or virological evidence of infection | 0 |
| Ambulatory | No activity limitation | 1 |
|  | Activity limitation | 2 |
| Hospitalized with mild disease | No oxygen therapy | 3 |
|  | Oxygen mask or nasal prongs | 4 |
| Hospitalized with serious disease | Non-invasive mechanical ventilation or high-flow nasal cannula | 5 |
|  | Intubation and invasive mechanical ventilation | 6 |
|  | Intubation and invasive mechanical ventilation + additional support such as pressors or extracorporeal membrane oxygenation | 7 |
| Death | Death | 8 |

# Cumulative illness rating score

| **ORGAN-SYSTEM** | **SEVERITY** |
| --- | --- |
| 1. Cardiac | 0-1-2-3-4 |
| 2. Vascular | 0-1-2-3-4 |
| 3. Hematological | 0-1-2-3-4 |
| 4. Respiratory | 0-1-2-3-4 |
| 5. Ophthalmological and ear, nose, and throat | 0-1-2-3-4 |
| 6. Upper gastrointestinal | 0-1-2-3-4 |
| 7. Lower gastrointestinal | 0-1-2-3-4 |
| 8. Hepatic and pancreatic | 0-1-2-3-4 |
| 9. Renal | 0-1-2-3-4 |
| 10. Genitourinary | 0-1-2-3-4 |
| 11. Musculoskeletal and cutaneous | 0-1-2-3-4 |
| 12. Neurological | 0-1-2-3-4 |
| 13. Endocrine, metabolic, mammary | 0-1-2-3-4 |
| 14. Psychiatric | 0-1-2-3-4 |

Score, depending on the extent to which the organ/system is affected: 0, absence of disease; 1, mild; 2, moderate; 3, severe; 4, very severe.

Adapted from Linn BS, Linn MW, Gurel L. Cumulative illness rating scale. J Am Geriatr Soc 1968; 16: 622-626.

# Concomitant treatments

Concomitant treatments are those treatments administered to both groups according to the conditions set out below.

## Standard of care for COVID-19 pneumonia

Treatment followed the national recommendations of the Spanish Agency of Medicines and Medical Devices (www.aemps.gob.es/la-aemps/ultima-informacion-de-la-aemps-acerca-del-covid%e2%80%9119/ ) and of the WHO (www.who.int/health-topics/coronavirus#tab=tab_1), which were regularly updated and in force during the inclusion period.

### Oxygen therapy

Nasal prongs, facemask, non-rebreather facemask, noninvasive mechanical ventilation, invasive mechanical ventilation (IMV) with orotracheal intubation, and extracorporeal membrane oxygenation (ECMO) if available.

### Prophylaxis or treatment of bacterial pulmonary superinfection

A third-generation cephalosporin (ceftriaxone, cefixime) should be prescribed. Doxycycline should be prescribed in the case of allergic patients.

• Dose and duration:

Ceftriaxone 1-2 g iv/24 hours for 5 days.

Cefixime 400 mg orally (po)/24 hours for 5 days.

Doxycycline 100 mg po-iv/12 hours for 5 days.

The oral route should be preferred when possible.

### Remdesivir

In patients hospitalized with SARS-CoV-2 and oxygen saturation ≤94% in room air who require supplementary oxygen or mechanical ventilation or ECMO.

• Dose and duration:

Patients who require IMV and/or ECMO: Loading dose administered on day 1 at 200 mg iv followed by a maintenance dose of 100 mg iv daily from day 2 to day 10.

Patients who require IMV and/or ECMO: Loading dose administered on day 1 at 200 mg IV followed by a maintenance dose of 100 mg iv daily from day 2 to day 5 (this dose can be extended a further 5 days, that is, until day 10, if the patient’s condition does not improve).

### Dexamethasone

Patients hospitalized with COVID-19 pneumonia.

• Dose and duration:

6 mg iv or po for 5 days.

## Treatment with corticosteroids

• Criteria for indication:

- At least 7 days since the onset of symptoms.

- At least 3 days with the specific treatment for each group (CsA+SOC vs. SOC), except when the severity of the patient’s condition requires corticosteroids to be administered earlier.

- Rule out superinfection.

- Radiological worsening with respect to baseline and/or need for oxygen therapy: FiO2 must be higher than the previous value in order to maintain saturation >92% (a saturation of 90-92% is equivalent to a PO2 of 60).

- Clinical-analytical worsening with respect to baseline, with at least 2 of the following values recorded:

 Lymphopenia <800/mm3

 Ferritin >500 ng/mL

 D-dimer ≥500-1000 ng/mL

 CRP >40 mg/L

 Increase in IL-6 ≥40 pg/mL

• Dose and duration:

- First, methylprednisolone bolus 250 mg iv every 24 hours for 3 days.

- Second, methylprednisolone 40 mg iv every 12 hours for 2 days, followed by 40 mg every 24 hours for 2 days, then discontinue.

## Biologics

Any patient can receive biologics if necessary, according to the indications. If the patient has IL-6 ≥40 pg/mL, then tocilizumab or sarilumab should be administered; if levels of IL-6 are <40 pg/mL, then anakinra should be administered.

### Tocilizumab or sarilumab

• Criteria for indication:

- At least 3 days with the specific treatment for each group (CsA+SOC vs. SOC).

- Failed treatment with corticosteroids, that is, no improvement in symptoms.

- Superinfection ruled out.

- Radiological worsening with respect to baseline and/or need for oxygen therapy: FiO2 must be higher than the previous value in order to maintain saturation >92% (a saturation of 90-92% is equivalent to a PO2 of 60).

- Clinical-analytical worsening with respect to baseline, with at least 2 of the following values recorded:

 Lymphopenia <800/mm3

 Ferritin >500 ng/mL

 D-dimer ≥500-1000 ng/mL

 CRP >40 mg/L

 Increase in IL-6 ≥40 pg/mL

• Tocilizumab and sarilumab are not recommended in the following cases:

- AST/ALT values higher than 10 times the upper limit of normal.

- Neutrophils <500/mm3.

- Platelets <50,000/mm3.

- Sepsis caused by pathogens other than SARS-CoV-2.

- Comorbid conditions that, in the opinion of the attending physician, can be indicative of a poor prognosis.

- Complicated diverticulitis or intestinal perforation.

- Ongoing skin infection (eg, pyoderma that is not controlled with antibiotic therapy).

• Dose and duration:

- Tocilizumab: 600 mg in patients weighing ≥75 kg and 400 mg in those weighing <75 kg (single dose). Exceptionally, a second infusion can be considered 12 hours after the first one in patients whose laboratory values rebound after a partial improvement, whether in terms of symptoms or laboratory values or both. The possibility that the rebound is due to a secondary infection (eg, subcutaneous infection or intestinal perforation) or other causes should be ruled out. In this sense, it is also recommended not to consider the increase in IL-6 levels as a reference parameter, since the mechanism of action of tocilizumab (IL-6 receptor blocker) leads us to expect that IL-6 levels would increase after administration of the drug.

- Sarilumab: 400 mg iv in a single infusion (2 vials of 200 mg diluted in 100 mL to be infused in 1 hour).

The use of tocilizumab or sarilumab depends on available pharmacy stock at Hospital Universitario Infanta Sofía.

### Anakinra

• Criteria for indication:

- At least 3 days with the specific treatment for each group (CsA vs. no treatment).

- Failure of corticosteroids, that is, the patient's symptoms do not improve.

- Superinfection ruled out.

- Radiologic worsening compared with baseline and/or need for oxygen therapy with an FiO2 greater than the previous value in order to maintain saturations at >92% (a value of 90-92% is equivalent to a PO2 of 60).

- Worsening of clinical and laboratory findings compared with baseline, with at least 2 of the following:

 Lymphopenia <800/mm3

 Ferritin >500 ng/mL

 D-dimer ≥500-1000 ng/mL

 CRP >40 mg/L

 IL-6 levels <40 pg/mL

• Anakinra is not recommended in the following cases:

- AST/ALT values above 10 times the upper limit of normal.

- Neutrophils <1,500/mm3.

- Platelets <50,000/mm3.

- Sepsis caused by pathogens other than SARS-CoV-2.

- Comorbidity that, according to the attending physician, could be indicative of a poor prognosis.

- Ongoing cutaneous infection (eg, pyoderma not controlled with antibiotics).

• Dose and duration:

100 mg sc/6 hours for 3 days.

Given that tocilizumab, sarilumab, and anakinra are registered by the AEMPS for the indications set out in their respective summaries of product characteristics, we refer to these documents for contraindications, warnings, special precautions, adverse reactions, and drug-drug interactions. These indications were all taken into account in the inclusion and exclusion criteria of the present study.

## Thromboprophylaxis

Thromboprophylaxis should be with low-molecular-weight heparin for all patients except those with active bleeding or platelets <30,000.

• Dose and duration:

- Patients with a high thrombotic risk:

<80 kg: 40 m/24 h (if eGFR <30, 20 mg/24 h).

80-100 kg: 60 mg/24 h (if eGFR <30, 20 mg/24 h).

>100 kg: 40 mg/12 h (if eGFR <30, 20 mg/12 h).

- Patients with a high risk of thrombosis defined as the following:

 Severe infection that fulfills at least 3 of the following: CRP >150, D-dimer >1,500, IL-6 >40, ferritin >1,000, lymphopenia <800.

 D-dimer ≥6 times the reference value (≥3,000 ng/mL).

 Patients with a sepsis-induced coagulopathy score ≥4 or disseminated intravascular coagulation score ≥5.

1 mg/kg/24 h (if eGFR <30, 0.5 mg/kg/24 h). Doses can be scaled down to prophylactic doses once symptoms and laboratory values have improved.

- Maintain until 10 days after discharge in all cases.

# Figures

**Figure 1. Time from diagnosis to methylprednisolone.**


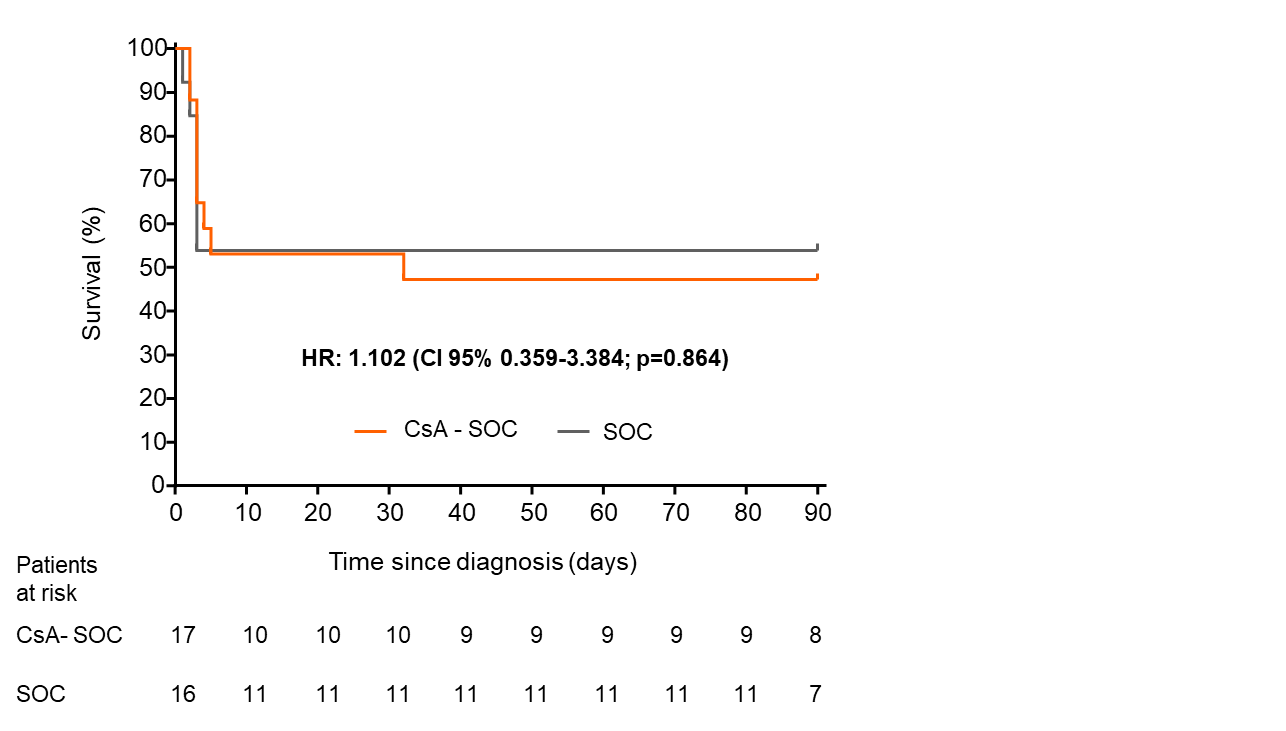


HR: hazard ratio; CI: confidence interval; CsA: cyclosporine A; SOC: standard of care.

**Figure 2. Time from diagnosis to biological therapy.**


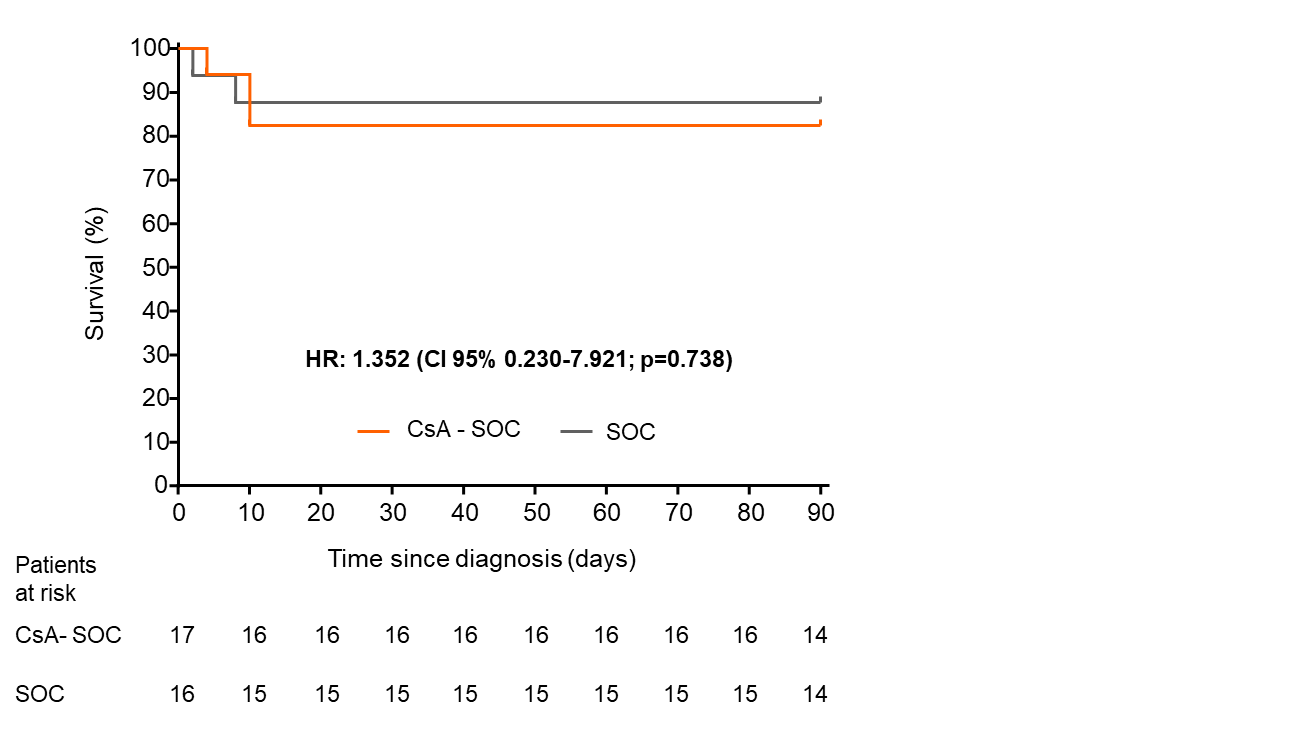


HR: hazard ratio; CI: confidence interval; CsA: cyclosporine A; SOC: standard of care.

**Figure 3. Time from diagnosis to adverse event.**


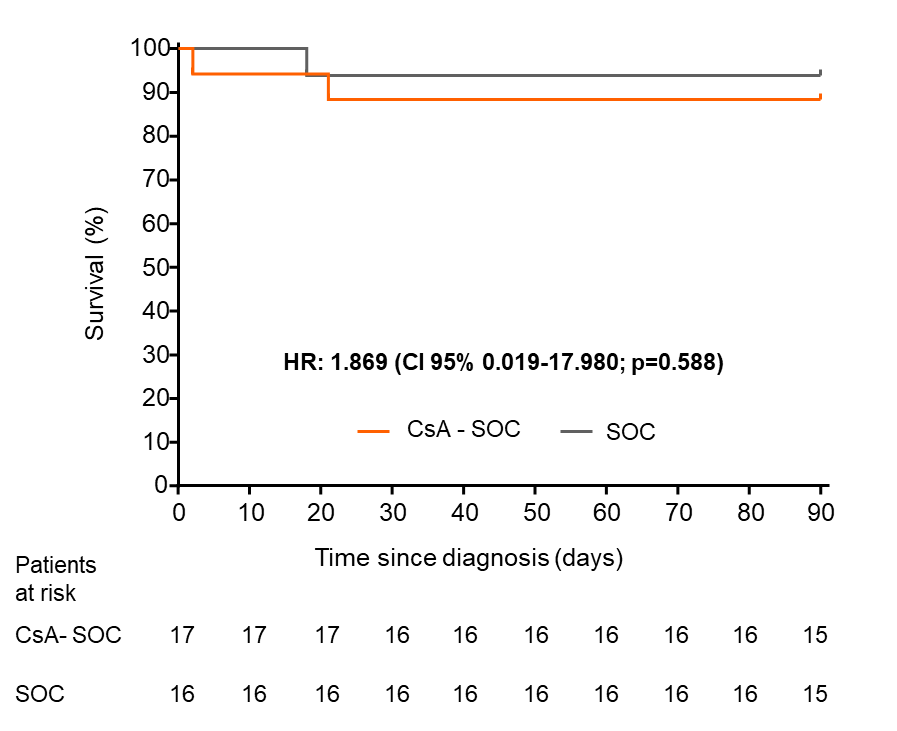


HR: hazard ratio; CI: confidence interval; CsA: cyclosporine A; SOC: standard of care.

# CONSORT 2010 checklist of information to include when reporting a pilot or feasibility trial*

| **Section/Topic** | **Item No** | **Checklist item** | **Reported on page No** |
| --- | --- | --- | --- |
| **Title and abstract** | | | |
|  | 1a | Identification as a pilot or feasibility randomised trial in the title | Page 1 |
|  | 1b | Structured summary of pilot trial design, methods, results, and conclusions (for specific guidance see CONSORT abstract extension for pilot trials) | Page 2 |
| **Introduction** | | | |
| Background and objectives | 2a | Scientific background and explanation of rationale for future definitive trial, and reasons for randomised pilot trial | Page 3 |
|  | 2b | Specific objectives or research questions for pilot trial | Page 3 |
| **Methods** | | | |
| Trial design | 3a | Description of pilot trial design (such as parallel, factorial) including allocation ratio | Page 3 |
|  | 3b | Important changes to methods after pilot trial commencement (such as eligibility criteria), with reasons |  |
| Participants | 4a | Eligibility criteria for participants | Page 3 |
|  | 4b | Settings and locations where the data were collected | Page 3,4 |
|  | 4c | How participants were identified and consented | Page 4 |
| Interventions | 5 | The interventions for each group with sufficient details to allow replication, including how and when they were actually administered | Page 4 |
| Outcomes | 6a | Completely defined prespecified assessments or measurements to address each pilot trial objective specified in 2b, including how and when they were assessed | Page 5 |
|  | 6b | Any changes to pilot trial assessments or measurements after the pilot trial commenced, with reasons |  |
|  | 6c | If applicable, prespecified criteria used to judge whether, or how, to proceed with future definitive trial |  |
| Sample size | 7a | Rationale for numbers in the pilot trial | Page 5 |
|  | 7b | When applicable, explanation of any interim analyses and stopping guidelines |  |
| Randomisation: |  |  |  |
| Sequence  generation | 8a | Method used to generate the random allocation sequence | Page 4 |
|  | 8b | Type of randomisation(s); details of any restriction (such as blocking and block size) | Page 4 |
| Allocation  concealment  mechanism | 9 | Mechanism used to implement the random allocation sequence (such as sequentially numbered containers), describing any steps taken to conceal the sequence until interventions were assigned | Page 4 |
| Implementation | 10 | Who generated the random allocation sequence, who enrolled participants, and who assigned participants to interventions | Page 4 |
| Blinding | 11a | If done, who was blinded after assignment to interventions (for example, participants, care providers, those assessing outcomes) and how | Page 4 |
|  | 11b | If relevant, description of the similarity of interventions |  |
| Statistical methods | 12 | Methods used to address each pilot trial objective whether qualitative or quantitative | Page 5-6 |
| **Results** | | | |
| Participant flow (a diagram is strongly recommended) | 13a | For each group, the numbers of participants who were approached and/or assessed for eligibility, randomly assigned, received intended treatment, and were assessed for each objective | Page 6, Figure 1 |
|  | 13b | For each group, losses and exclusions after randomisation, together with reasons | Figure 1 |
| Recruitment | 14a | Dates defining the periods of recruitment and follow-up | Page 6 |
|  | 14b | Why the pilot trial ended or was stopped |  |
| Baseline data | 15 | A table showing baseline demographic and clinical characteristics for each group | Table1 (page 16-17) |
| Numbers analysed | 16 | For each objective, number of participants (denominator) included in each analysis. If relevant, these numbers should be by randomised group | Table 2 (page 18), Table 3 (page 19), Table 4 (page 20), Page 6- 7 |
| Outcomes and estimation | 17 | For each objective, results including expressions of uncertainty (such as 95% confidence interval) for any  estimates. If relevant, these results should be by randomised group | Table 2 (page 18), Table 3 (page 19), Table 4 (page 20),  Page 6-8 |
| Ancillary analyses | 18 | Results of any other analyses performed that could be used to inform the future definitive trial |  |
| Harms | 19 | All important harms or unintended effects in each group (for specific guidance see CONSORT for harms) | Table 2 (page 18), Page 7-8 |
|  | 19a | If relevant, other important unintended consequences |  |
| **Discussion** | | | |
| Limitations | 20 | Pilot trial limitations, addressing sources of potential bias and remaining uncertainty about feasibility | Page 9-10 |
| Generalisability | 21 | Generalisability (applicability) of pilot trial methods and findings to future definitive trial and other studies | Page 9-10 |
| Interpretation | 22 | Interpretation consistent with pilot trial objectives and findings, balancing potential benefits and harms, and  considering other relevant evidence | Page 8-9-10 |
|  | 22a | Implications for progression from pilot to future definitive trial, including any proposed amendments | Page 9-10 |
| **Other information** | | |  |
| Registration | 23 | Registration number for pilot trial and name of trial registry | Page 2-3 |
| Protocol | 24 | Where the pilot trial protocol can be accessed, if available | Related files |
| Funding | 25 | Sources of funding and other support (such as supply of drugs), role of funders | Page 11 |
|  | 26 | Ethical approval or approval by research review committee, confirmed with reference number | Page 3 |

Citation: Eldridge SM, Chan CL, Campbell MJ, Bond CM, Hopewell S, Thabane L, et al. CONSORT 2010 statement: extension to randomised pilot and feasibility trials. BMJ. 2016;355.

*We strongly recommend reading this statement in conjunction with the CONSORT 2010, extension to randomised pilot and feasibility trials, Explanation and Elaboration for important clarifications on all the items. If relevant, we also recommend reading CONSORT extensions for cluster randomised trials, non-inferiority and equivalence trials, non-pharmacological treatments, herbal interventions, and pragmatic trials. Additional extensions are forthcoming: for those and for up to date references relevant to this checklist, see www.consort-statement.org.
